# The effect of empowerment on the self-efficacy, quality of life and clinical and laboratory indicators of patients treated with hemodialysis: a randomized controlled trial

**DOI:** 10.1186/1477-7525-10-115

**Published:** 2012-09-20

**Authors:** Marzieh Moattari, Marzieh Ebrahimi, Nasrin Sharifi, Jamshid Rouzbeh

**Affiliations:** 1 Fatemeh (pbuh) School of Nursing & Midwifery, Shiraz University of Medical Sciences (SUMS), Zand Blvd, P.O. Box 71345-1359, Shiraz, 71936-13119, Iran; 2 Shiraz Nephrology Urology Research Center, Shahid Faghihi Hospital, Shiraz University of Medical Sciences (SUMS), Shiraz, Iran

**Keywords:** Hemodialysis, Empowerment, Quality of life, Clinical indicators, Self-efficacy

## Abstract

**Background:**

Hemodialysis patients face numerous physical and psychological stresses that result in reduced health. The aim of this study is to determine the impact of an empowerment program on self-efficacy, quality of life, clinical indicators of blood pressure and interdialytic weight gain, and laboratory results in these patients.

**Methods:**

This randomized, controlled trial was conducted at Boo Ali Sina Dialysis Center, Shiraz, Iran. A total of 48 hemodialysis patients participated in this study. After acquisition of informed consent, eligible patients were randomly divided into two groups, control and experimental. Pre-test data were obtained by using a demographic data form and two questionnaires for self-efficacy and quality of life. Blood pressure and interdialytic weight gain were measured. We extracted laboratory data from patients’ charts. A six-week empowerment intervention that included four individual and two group counselling sessions was performed for the experimental group. Six weeks after intervention, post-test data were obtained from both groups in the same manner as the pre-test. Data were analyzed by ANCOVA using SPSS v11.5.

**Results:**

There were no statistically significant differences in demographic variables between the groups. Pre-test mean scores for self-efficacy, quality of life, blood pressure, interdialytic weight gain and laboratory results did not differ between the groups. There was a significant difference between the experimental and control groups in terms of pre-to post-intervention changes in overall self-efficacy scores, stress reduction, and decision making, in addition to overall quality of life and all dimensions included within quality of life based on this questionnaire. Additionally, the pre- to post-intervention changes in systolic/diastolic blood pressures, interdialytic weight gain, hemoglobin and hematocrit levels significantly differed between the groups.

**Conclusion:**

Our study demonstrates that a combination of individual and group empowerment counselling sessions improves self-efficacy, quality of life, clinical signs, and hemoglobin and hematocrit levels in hemodialysis patients. Empowerment of hemodialysis patients should be considered in hemodialysis centers to assist patients with the management of their health-related problems.

**Trial registration:**

Irct ID: IRCT138901172621N4

## Introduction

Dialysis lengthens the life of patients with end stage renal disease (ESRD) [1]. In 2001, it was predicted that by 2010, the numbers of patients with ESRD would reach 129,200 ± 7742 new patients, 651,330 ± 15874 long-term ESRD patients, and 520,240 ± 25609 patients on hemodialysis (HD) [2]. In 2007, there were 29000 people in Iran with CRF, of whom 14000 were treated with HD [3]. HD patients experience many physical and psychological problems. Zamanzadeh et al. have conducted a correlational study that measured the quality of life (QoL) and social support of 164 HD patients. The researchers measured QoL by utilizing a combination of three different questionnaires following validity and reliability approval. The measurement of social support also included the combination of an additional three valid, reliable questionnaires. In this study, the authors have not explained their rationale for combining different questionnaires to measure these variables. However they have reported that over 50% of patients experienced physical and psychological problems. These aspects of QoL were associated with social support [4]. Curtin et al., in a cross-sectional study measured self-management and knowledge in 372 patients from 17 dialysis centers and determined that patients were low self-managers. The most common self-management strategies used by patients were the cooperative/participatory activities of self-care during HD and shared responsibility in care with medical personnel. Another important finding of their study was the positive association between patient knowledge of kidney disease and treatment to mental health functioning as measured by the SF-12 Mental Component Summary [5]. In another study, Sajjadi et al. have highlighted the necessity of constant participation of HD patients in self-care activities to alleviate problems associated with the illness and its treatment. They conducted a randomized controlled trial with 60 HD patients to elucidate the effects of self-care education based on needs assessment on depression. The study intervention consisted of two, 45 minute self-care education programs in addition to an educational booklet for the experimental group. Patient depression was measured by a short self-reporting scale. Although this scale is more appropriate for measurement of depression in the general population, research has supported the effectiveness of this self-care education program in reducing depression amongst HD patients [6].

It appears that there is an increased likelihood for HD patients to engage in self-management if their self-care self-efficacy is improved by empowerment programs. An association has been shown between higher perceived self-efficacy scores and QoL [7], increased communication, partnership, self-care, and medication-adherence behaviors [8]. Empowerment of patients is a model of intervention used to facilitate decision making and self-care [9]. The components of this model include self-management education [10], enhancement of goal setting ability, problem solving, stress management, social support and motivation [11].

While the empowerment of patients who suffer from chronic disease such as diabetes have been considered in different studies [12-15], few studies have been performed to determine the possible effects of empowerment in HD patients. Studies on HD patients are mostly descriptive or correlational, highlighting patient behavior and knowledge [5,8,16] or confirming the correlations between age, employment status, dialysis modality, and length of dialysis to knowledge expectations [17]. The associations between altered QoL to physical and psychiatric co-morbidities, in particular depression and anxiety, of patients undergoing chronic HD have been confirmed in another study [18]. In a systematic review conducted in 2008 on educational interventions in kidney disease care, promising results were noted for dialysis educational interventions aimed at improving dialysis and/or fluid concordance, exercise, and coping/adaptation. Unfortunately many of these studies lacked rigorous evaluation [19]. Only one of the reviewed articles in this systematic review contained the word “empowerment”. The article of interest was a randomized controlled trial that researched the effectiveness of an empowerment program on empowerment level, self-care self-efficacy and depression in patients with ESRD. This study randomly allocated 50 qualified patients to empowerment and control groups. A problem solving approach was used in the study’s intervention that included identification of problems, exploration of emotions, setting goals and appropriate strategies to overcome problems, creation and implementation of behavioral change plans, and stress management. Findings of this study showed significant improvements in empowerment, self-care self-efficacy and depression [20]. In an intervention-evaluation design the effectiveness of using the empowerment concept during the development of a mutual-help group for HD was investigated. The intervention process was conducted in four phases: (1) assessment, (2) planning, (3) action and (4) evaluation/feedback. A mutual-help group was formed that met eight times for group activities over a three-month study period. Activities such as exchange of experiences, knowledge of self-care, preparation for travel, educational seminars, social welfare and artistic skills such as drawing and calligraphy were implemented. The results showed a significant reduction in physical symptoms and improvements in social support and QoL [21].

A variety of educational interventions have been implemented in various studies. Different outcomes are expected in these studies because of the differences between the intervention and expected or measured outcomes that result from the empowerment programs. This might be due to the fact that empowerment differs across people, contexts, and times and could be considered as a nomological network, which includes intrapersonal, interactional, and behavioral components [22]. For example, in one study it was concluded that the internal locus of control as a component of patient characteristics was positively associated with the mental QoL component, in particular, the mental health score [18]. Additionally, it is a belief that nurses’ perceptions of what constitutes quality nursing care may influence their care of the person receiving HD [23]. Other factors such as age, disease stages, patient characteristics, co-morbidity, available social supports for patients, as well as background, attitudes and expertise of nurses make each nurse-patient relationship a unique experience. Thus, there is no unified educational prescription for different patients, or in other words, one size does not fit all.

Empowering HD patients to solve their own problems and take responsibility for managing their illness and its associated problems or complications through a problem solving approach, education and support based on an individual patient’s needs and shared goal setting can lower the limitations of providing the same protocol for all. There is a lack of sufficient literature to support the effectiveness of empowerment programs on QoL, clinical and laboratory indicators in HD patients. This is noteworthy in the context of Iranian culture and particularly regarding HD patients, thus this study aims to investigate the effect of empowerment on self-care self-efficacy, QoL, and clinical and laboratory indicators in a group of Iranian ESRD patients.

## Methods

This study was a parallel groups, randomized clinical trial that assigned eligible patients undergoing HD by the simple randomization method on a 1:1 ratio to receive either usual care (control, n = 25) or an empowerment program (experimental, n = 25).We approached potential participants at Boo-Ali Sina Dialysis Center, Shiraz, Fars Province, Iran, which is the largest dialysis center in the Middle East over a three month period, from Nov 2009 to Feb 2010. There was no patient phone contact, as all enrolled patients were routinely seen at Boo-Ali Sina Dialysis Center as part of their routine HD.

Eligibility criteria included the following: subjects diagnosed with ESRD and treated with HD for at least three months, ages 18 – 60 years, patients who lived at home, were able to read and write, had no psychiatric or cognitive disorders, and were willing to participate in the study. Those with acute illnesses or hospitalized were excluded.

Patients in the experimental group completed a six-week empowerment program that consisted of four individual and two group counselling sessions. During individual counselling patients were assisted with the development of necessary skills and self-awareness in goal setting and problem solving. Patients were evaluated by two different assessment forms, those treated with HD for three months to less than one year or those treated with HD for more than one year. The forms were downloaded from http://www.lifeoptions.org and were used to identify patients’ problem areas for self-management and highlight their educational needs in ten different aspects that included: medical condition, relationship with family and friends, problems associated with work, school and insurance, eating, future, feelings, responsibilities, life style, everyday activities, and relationship with staff. Emotions associated with these problems such as loneliness, feelings of isolation, hopelessness, fatigue, and suicidal ideation were explored, and a mutual goal setting approach was developed to overcome these problems. A written agreement was made between the nurse and patient regarding the behavior change plan during the intervention with the intent to increase the likelihood of patients taking the necessary actions. A behavior change plan was based on the patient’s priority. Self-efficacy in regards to each behavior change plan was assessed by a visual analog scale. Patients’ families were involved in the process of empowerment at the patient’s request. Patients were informed about available social support and were referred to the appropriate centers and experts if necessary. All individual sessions were conducted by the second author who is qualified to run these sessions and address HD patients’ problems. During individual sessions each patient was provided feedback regarding their clinical indicators and laboratory tests.

To run group counselling, the intervention group was divided into two smaller groups, each attending two sessions for 1.5 - 2 hours. These sessions were conducted by a psychiatric nurse and focused on stress management, problem-focused and emotion-focused coping strategies, social support and motivation. Group discussion/reflection based on live patients’ experiences, role playing, and question and answer techniques were used for making patients aware of their own and others coping strategies and problem solving techniques. Muscle relaxation exercise was practiced in the group and a muscle relaxation audiotape was given to the patients for stress management. Patients were contacted by phone to facilitate their continuous involvement in the intervention. The empowerment program was offered only to the intervention group and the control group received the usual treatment provided in the setting.

### Outcome measures

Primary outcome measures were self-care self-efficacy and QoL. Secondary outcomes were interdialytic weight gain (IDWG), blood pressure, and laboratory tests results that included sodium (Na^+^), potassium (K^+^), creatinine (Cr), blood urea nitrogen (BUN), phosphorous (P), calcium (Ca^+^), hemoglobin and hematocrit (H&H).

Outcomes were measured prior to treatment (baseline) and at six weeks post-intervention. We measured self-care self-efficacy and QoL by the Strategies Used by People to Promote Health (SUPPH) and QoL questionnaires. Questionnaires were completed by patients under the supervision of a nurse who was blinded to treatment allocation. IDWG and blood pressure were measured by a trained nurse who was also blinded to the treatment allocation. Laboratory tests results for Na^+^, K^+^, Cr, BUN, P, Ca^+^, and H&H were retrieved from the patients’ charts.

Sample size was estimated based on the findings (d = 25; pooled sd = 30) of another study regarding QoL [9]. We enrolled 50 patients with the intent to obtain sufficient statistical power (80%) in detecting differences between the groups and to predict the study outcomes between both groups with a significance of p <0.05. In the post-test, 2 patients failed to continue participation in the control group; one changed to another dialysis center and the other patient underwent a kidney transplant. Therefore data analyses were performed on 48 patients.

### Instruments

Demographic data that included age, sex, marital status, education level, as well as the duration of treatment with HD and frequency of HD per week were gathered using a locally designed data form.

Self-care self-efficacy was measured by a 29-item self-reported questionnaire. The first version of this questionnaire (SUPPH) was developed based on the self-efficacy theory and includes four dimensions: coping, stress, decision making, and enjoying life. In another study dimensions of the instrument were reduced to three: positive attitudes (16 questions), stress (10 questions), and decision making (3 questions) [24]. The latter questionnaire was translated into Persian (Farsi) and back-translated into English to ensure its validity, which was further confirmed by a panel of experts. Next, the instrument was distributed to 30 representative patients for measurement of reliability. Cronbach’s alpha for the overall questionnaire was 0.91 and for the dimensions of the instrument, it was as follows: stress reduction (0.79), decision making (0.8), and positive attitude (0.87). Reliability of the original form in these dimensions was reported as 0.89 (stress reduction), 0.83 (decision making), and 0.92 (positive attitude) [25]. Possible scores for the overall self-care self-efficacy questionnaire were 29 - 145. The ranges for the dimensions were: positive attitude (16 - 80), stress (10 - 50), and decision making (3 - 15). Higher scores reflect better outcomes.

We used the QoL questionnaire that was developed in 1984 by Carol Estwing Ferrans and Marjorie Powers [26]. The instrument consists of two parts. The first part measures satisfaction with various aspects of life and the second measures the importance of the same aspects. Importance ratings are used to weigh the satisfaction responses. Therefore, the scores reflect the respondents’ satisfaction with the aspects of life as they value. Items rated as more important have a greater impact on scores than those of lesser importance. The scores are calculated both for overall QoL and four subscales of health and functioning; psychological/spiritual; social and economic; and family.

The QoL can be used either as a self-administered questionnaire or through an interview. If self-administered, the QoL takes approximately ten minutes to complete. No special training is required.

The validity and reliability of this questionnaire was reported by Ferrans and Powers in 1985 and 1992 [27,28]. This questionnaire was translated into Persian by Rambod and its validity and reliability have been confirmed [3]. The possible range for the final scores is the same for all four subscales and for the overall (total) score. Total scores may be classified in three levels: desirable (score: 20 - 30), relatively desirable (score: 10 - 19) and unfavorable (score: 0 - 9).

Pre-dialysis blood pressure measurements were taken using a mercury sphygmomanometer on the non-fistula arm in accordance with the routine university protocol. In two consecutive sessions three sitting blood pressure measurements were taken ten minutes apart, after the patient had been resting in a quiet room for at least five minutes. The average of the last two measurements in two consecutive HD sessions (four readings) was utilized as the standardized blood pressure for this study.

The amount of weight gain between the end of one dialysis session and the beginning of the next (IDWG) was used as a valid marker for adherence to fluid restriction/regulation. Weight gain was measured in three consecutive HD sessions and the mean was used for analysis.

All patients were registered at the main center in which the study was conducted and all of their tests were performed in the same center. This center is affiliated with the Shiraz University of Medical Sciences (SUMS) and follows the university standards and protocols.

### Ethical considerations

Institutional Review Board (IRB) approval for the study was obtained from the Ethics Committee of Shiraz University of Medical Sciences (ECSUMS). Written consent was obtained from each patient. The purpose of study, voluntary participation, confidentiality and freedom to discontinue at any time without being left untreated was reviewed with patients prior to their participation.

### Data analysis

**Figure 1:**
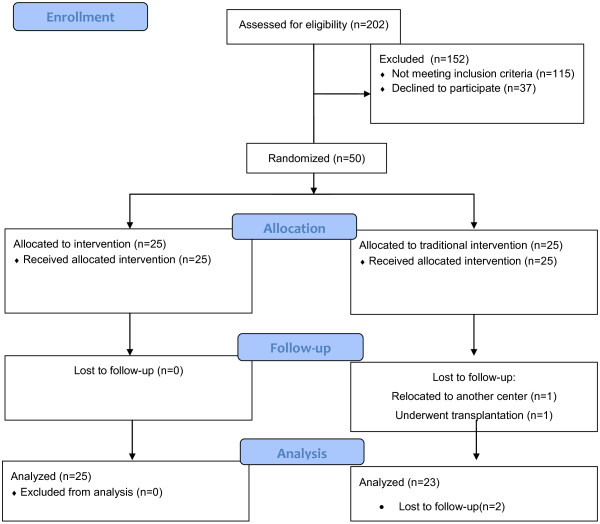
Consolidated Standards of Reporting Trials ( CONSORT) 2010 Study Flow Diagram.

Descriptive and inferential statistics that included frequency, percentage, mean, standard deviation, and ANCOVA were used by SPSS v11.5. All 25 patients in the treatment group and 23 out of 25 patients in the control group were included in the analysis (Figure 1). Self-efficacy data was analyzed based on its three dimensions. QoL data was analyzed based on the guideline provided by Ferrans and Power using the statistical program Syntax for SPSS-PC based manual analysis [26]. Based on the guideline, the analysis should be performed through four stages of: recoding satisfaction scores, weighting satisfaction responses with the paired importance responses, obtaining preliminary sum for the overall (total) score, and obtaining final overall (total) score. The possible range for the final scores is the same for all four subscales and for the overall (total) score. Total scores may be classified in three levels: desirable (score: 20 - 30), relatively desirable (score: 10 - 19) and unfavorable (score: 0 - 9).

## Results

The results of this study are based on the analyses of 48 out of 50 patients involved in the study (Figure 1).

### Patient characteristics

There were 31 (64.6%) male patients. Patients had a mean age of 38.16 years, of whom 60.4% were married. Their education level was as follows: elementary (12.5%), middle school (39.6%), high school (29.2%) and university educated (18.7%). The mean duration of HD was 30.16 months [range: 3 - 156 months (13 years)]. HD was administered twice weekly in 27.1% of cases and 72.9% received HD three times a week. Demographic variables for the study population are shown in Table 1.

**Table 1:** Demographic variables of study population*


**Demographic variables**	**Control group**	**Experimental group**
Mean age (yrs) at study entry ± SD	37.3 ± 12.79	38.56 ± 11.4
Sex (n = 48) Males (%)	16 (69.6)	15 (60)
Married (%)	15 (65.2 )	15 (60)
Educational status (%)		
Primary school	4 (17.4)	2 (8)
Secondary school	8 (34.8)	11 (44)
Post-secondary school diploma	6 (26.1)	8 (32)
Undergraduate degree	5 (21.7)	4 (16)
Dialysis (%)		
Twice per week	5 (21.7)	8 (32)
Three times per week	18 (78.3)	17 (68)
Renal risk markers (%)		
Hypertension	7 (30.4)	6 (24)
Diabetes	5 (21.7)	4 (16)
Renal stones	4 (17.4)	0
Infection	1 (4.3)	3 (12)
Other	6 (26.1)	12 (48)

*All comparisons are non-significant. p > 0.05.

### Effect of intervention on self-efficacy and quality of life (QoL)

Adjusted mean scores for self-efficacy and QoL in both groups and between groups adjusted mean difference are shown in Table 2. Based on the results of ANCOVA, a significant change was observed between the groups in terms of stress reduction as well as the decision making dimension and overall self-efficacy scores. Also, a significant difference was found in the overall mean score of QoL and in all dimensions of the QoL between the groups.

**Table 2:** Adjusted mean scores of self-efficacy and QoL in both groups and between groups adjusted mean difference


**Variables**	**Adjusted mean (SD) Control Experimental**		**Adjusted mean difference (95%CI)**	**p-value**
Stress reduction	29.94 ± 5.22	33.54 ± 5.2	−3.6 (-4.4 to -1.57)	<0.02
Decision making	7.66 ± 2.01	10.87 ± 2.05	−3.21 (-4.39 to-2.03)	<0.001
Positive attitude	49.12 **±** 7.71	53.21 **±** 7.75	−4.09 (-8.59 to 0.41)	>0.073
Total self-care self-efficacy score	85.78 **±** 11.16	97.8 ± 11.15	−12.02 (-18.41 to -5.53)	<0.001
Health and functioning	15.78 **±** 2.97	19.27 ± 3.00	−3.49 (-5.23 to -1.75)	<0.001
Social and economic	16.25 **±** 2.94	19 ± 2.95	−2.75 (-4.46 to -1.04)	<0.002
Psychological and spiritual	18.76 **±** 3.69	20.94 ± 3.69	−2.18 (-4.33 to -0.03)	<0.047
Family	23.36 ± 4.5	26.41 ± 4.5	−3.05 (-5.66 to -0.420a)	<0.024
Total quality of life score	17.54 ± 2.51	20.47 ± 2.5	−2.93 (-4.39 to -1.47)	<0.001

### Effects of intervention on clinical and laboratory data

Results regarding blood pressure, IDWG and laboratory tests are shown in Table 3. Adjusted mean differences between the two groups are significant in both systolic and diastolic blood pressure and IDWG. No significant change was found between the groups in their laboratory results, with the exception of H&H.

**Table 3:** Adjusted mean values of clinical and lab indicators in both groups and between groups difference

**Indicators**	**Adjusted mean (sd)**		**Adjusted mean difference (95%CI)**	**p-value**
	**Control**	**Experimental**		
Systolic blood pressure (mm Hg)	143.37 **±** 11.56	129.02 **±** 11.58	14.35 (7.62 to 21.07)	<0.001
Diastolic blood pressure (mm Hg)	83.1 **±** 6.08	77.48 **±** 6.08	5.62 (2.08 to 9.16)	<0.003
Interdialytic weight gain (kg)	2.52 **±** 0.7	2.08 **±** 0.71	0.44 (0.03 to 0.85)	<0.039
Hb (g/dl)	10.98 **±** 2.13	12.97 **±** 2.16	−1.99 (-3.24 to 0.74)	<0.005
Hct (%)	33.61 **±** 6.74	40.01 **±** 6.76	−6.4 (-10.33 to -2.47)	<0.004
BUN (mg/dL)	42.45 **±** 14.85	39.76 **±** 14.89	2.69 (-5.96 to 11.34)	>0.55
Cr (mg/dL)	7.48 **±** 1.84	7.24 **±** 1.87	0.24 (- 0.84 to 1.32)	>0.677
Na^+^ (mEq/L)	138.14 **±** 3.64	136.18 **±** 3.65	1.96 (-0.16 to 4.08)	>0.086
K^+^ (mEq/L)	5.35 **±** 0.9	5.11 **±** 0.91	0.24 (-0.29 to 0.77)	>0.393
Ca^+^ (mg/dL)	8.82 **±** 0.95	9.09 **±** 0.96	−0.27 (-0.83 to 0.29)	>0.352
P (mg/dL)	4.53 **±** 1.43	4.77 **±** 1.44	−0.24 (-1.07 to 0.59)	>0.59

Hb, Hemoglobin; Hct, Hematocrit; BUN, Blood urea nitrogen; Cr, Creatinine; Na^+^, Sodium; K^+^, Potassium; Ca^+^, Calcium; P, Phosphorous.

## Discussion

This study aimed to investigate the effect of empowerment on self-care self-efficacy, QoL, and clinical and laboratory indicators in a group of ESRD patients. Results of the study supported the beneficial effects of the intervention on improvements in self-care self-efficacy, QoL, H&H, stabilizing blood pressure, and lowering IDWG. Numerous evidences have suggested that the empowerment of ESRD patients treated with HD are associated with outcomes such as improvements in QoL [5,11], facilitation of decision making, self-care [9], effective management of illness-related problems, and appropriate decision making [10]. These outcomes are somehow related to the concept of self-efficacy. However, self-efficacy has been referred to in different ways such as the goal of an empowerment-based intervention, indicator of empowerment, predisposing factor, and the acquisition of necessary skills for an empowerment process [29]. In this study, we have defined self-efficacy as the outcome of an empowerment program. However, in the current study, two components of self-efficacy (decision making and stress reduction) improved, whereas no improvement in positive attitudes was noted. Improvements in decision making and stress reduction can be attributed to the features of our empowerment program such as the provision of individual support for problem solving and engagement of family members and health care providers in the problem solving process. Additionally, patient participation in the group sessions during the study intervention and practiced relaxation techniques may be considered as reasons for the observed changes in stress reduction. Although we have no criteria available to judge the value of the minimally important difference (MID), but logically even slight changes in self-efficacy can have tremendous value because feelings of competency can have greatly impact a patients’ mental status. This, in turn, can affect their ability to overcome problems they encounter. Improved mental status releases expendable energy for problem solving. In the current study, the observed changes in decision making, stress reduction and overall self-efficacy scores are more than half of a SD as a landmark, which is a previously suggested [30] threshold of discrimination for changes in health-related quality of life for chronic diseases

Despite the observed change in the positive attitude score, which was more than what has been suggested as the minimum important difference (MID) between groups, [30,31] the difference between our study groups was not significant. Insignificant changes in positive attitude may be due to the complexity of the problems encountered by patients, which may require a longer intervention program. The change in attitude of HD patients toward their illness seems to be difficult processes as patients continually evaluate their condition, express feelings such as loneliness, isolation, hopelessness, fatigue, and even suicidal ideation. Regardless of these difficulties, the findings are important and require us to consider appropriate measures in our empowerment programs in future studies.

In the present study, the overall QoL mean score was relatively desirable which concurred with the findings of other studies [27,32]. However, several studies have reported low QoL amongst HD patients [33-35]. Walters and colleagues have concluded in their study that when dialysis treatment begins for ESRD patients, QoL is reduced [36].A negative linear correlation between QoL and duration of HD has been previously shown [32]. Therefore, taking appropriate measures such as education and patient empowerment has been taken into account in more depth.

A comparison between the experimental and control groups revealed a significant difference in overall mean score of the QoL and all of its dimensions. As previously mentioned, QoL and the scores of its dimensions can be categorized in three levels: desirable, relatively desirable, and unfavorable. In comparing the QoL measures in this study with what has been suggested for each category it can be stated that adjusted mean scores of the experimental group in terms of total QoL, as well as the psychological and spiritual, and family dimensions were at desirable levels. Scores for the other dimensions were approximately near to desirable. In the control group, all the QoL adjusted mean scores with the exception of the family dimension score were at the relatively desirable level. In a comparison of the lower boundary of confidence interval of total QoL (4.39) with the previously determined MID (3-4) in another study [37], we were able to draw a definitive conclusion regarding the treatment effect on QoL. The reason for these clinically and statistically significant differences between the two groups could be attributed to empowerment intervention and self-management education that was executed in the study. Due to their active participation in empowerment program, patients learned to take responsibility for solving health-related problems and self-care, and cooperated in management of their care. We have considered the cooperation between patients and nursing staff in identifying the patients’ problems as the key point in the process of empowerment [38]. However, due to the differences between the health care providers’ and patients’ perceptions of requirements as highlighted elsewhere [39], this was not an easy process. We should spend considerable time with patients to reach an agreement on problem identification and its priority, in addition to the necessary strategies for problem solving, implementing and evaluation of the action plan.

Of note, the scores of the family dimension component of QoL were elevated in both groups both in the pre- and post-test. This was possibly a result of the cultural context of Iranian families in which family members express concern about the patient’s problem and provide support for their loved one. In a study conducted in Iran, the most desired aspect of social support was emotional support. The most desired social support received by the patient was from their family (89%), while organizational support was the least desired [4]. Ferrans and Powers in a study conducted in a different culture reported that the QoL family dimension score was at a desirable level (20 to 30) [27].

The improved QoL score observed in this study was parallel to the findings of another study that used a mutual-support group [21]. However, theirs was an intervention-evaluation design study that did not have a control group, where all the sessions were conducted in a group setting. The design of our study was based on a randomized controlled trial in which the intervention was based on both individual and group consultations. Consultation and group intervention have been shown to improve QoL in HD patients [40,41].

Findings related to blood pressure supported the effectiveness of the empowerment program in maintaining both systolic and diastolic blood pressures in an acceptable range. In another study, a significant reduction in systolic and diastolic blood pressure was shown due to a diet education program [42]. The significant difference between the two groups found in our study was more related to the increase of blood pressure in the control group (ineffectiveness of conventional program) rather than a decrease of blood pressure in the experimental group. Of course, the latter was not expected as the mean pre-test blood pressure in both groups were about the normal range for chronic kidney disease patients (<130/80 mmHg) [43], and within the limit (<140/90 mmHg) suggested by the National Kidney Foundation for pre-dialysis [44]. It seemed that the comprehensive nature of our newly designed intervention was responsible for enabling patients (as was evident in their self-efficacy) to maintain their blood pressure within an acceptable range. It is believed that an ideal blood pressure in the HD patient would be associated with hemodynamic stability during dialysis, orthostatic tolerance after dialysis, better cardiovascular survival, and optimal health-related QoL [31]. However according to Saint-Remy and Krzesinski, blood pressure control in HD patients is not an easy task because extracellular volume modifications during and between the dialysis sessions can have a considerable and unpredictable effect on blood pressure [43].

Although measurement of blood pressure was done by a trained nurse, two points must be considered regarding reliability of the results. First, we measured pre-dialysis blood pressures, which have been reported to be biased estimates of systolic and diastolic blood pressure by a variable amount. Pre-dialysis measurements could be higher because of increased intravascular volume, withholding antihypertensive medications immediately prior to treatment, white coat syndrome, and lack of standardized measurements [45]. Secondly, the possibility of overestimation (50 mmHg) or underestimation (20 mmHg) of blood pressure as stated by Saint-Remy and Krzesinski existed. These researchers have cautioned medical professionals about the consideration of blood pressure measurements in the HD unit as quantitative and propose that such readings be a qualitative indicator of control (or lack of control) [43]. Ambulatory blood pressure monitoring or self-measurement by patients using home blood pressure monitoring has been suggested in different studies as reflective of a better validated measure of blood pressure [46,47]. However, regarding the suggested approaches for blood pressure control in HD patients, first by slow and smooth removal of extracellular volume (dry weight) and thereafter by appropriate antihypertensive medication [30], the possible effect of the intervention on medication adherence should not be ignored.

Our study showed that IDWG, as a clinical indicator, was affected by the empowerment program as the experimental group had lower IDWG. In a comparison of the adjusted mean IDWG of both groups to what has been reported in studies from the US (2.67 ± 1.39) and Germany (2.28 ± 1.12), [48] we may state that our control group IDWG was within these ranges, whereas the IDWG of our experimental group was lower than observed in the US and German studies, which was reflective of the effect of our intervention program. Measures exist for the correction of water retention between HD sessions that result in IDWG and edema following fluid and food intake. These include removal of water during dialysis either as an isolated treatment (ultrafiltration) or in combination with dialysis [49] and lowering dialysate (Na^+^) concentration [50]. As this was not the case in our study we attributed our results to the empowerment program of the study which enabled patients to enhance their adherence behavior and decision making in order to overcome the barriers of fluid intake restriction. In addition, feedback from individual sessions and awareness about strategies used by other patients in group sessions could be considered as motivating factors for patients to monitor their weights. As the considered minimally important IDWG in this study falls within the confidence interval, we cannot with certainty conclude that the empowerment program of the study was responsible for this change. The effect of education or motivational interviews on IDWG or improvement of adherence has been supported by other studies [43,51].

Analysis of laboratory results (Na^+^, K^+^, Cr, BUN, P, Ca^+^, and H&H) showed no significant difference between the two groups, with the exception of H&H which was significantly higher in the experimental group. That means the intervention program unintentionally increased hemoglobin levels, which was not in accordance with FDA (below 12 g/dl) recommendations [52]. The increase in H&H can be attributed to the empowerment program which was mostly based on the individual needs and approaching problem areas such as eating and life style. Different studies have reported an association between higher hemoglobin levels and increased mortality risk due to cardiovascular disease in both dialysis and non-dialysis chronic kidney disease patients [53]. However, the noted increase in the current study was not clinically significant because patients’ medical conditions were monitored by their physicians, who likely made some modifications in their treatment modalities such as erythropoietin-stimulating agent dose adjustments. Therefore it seemed that the empowerment program has the potential to lower the dosage of medication and therefore lower the cost of treatment. In another study, the beneficial effect of increasing hematocrit on QoL and its safety in selected HD patients has been reported [54].

Our study demonstrates that a combination of individual and group empowerment improves self-efficacy, QoL, clinical signs, and H&H levels in HD patients. Empowerment of HD patients should be considered in HD centers in order to assist patients with management of their health-related problems.

Due to the physical, social, psychological and cognitive complications of HD, nursing intervention based on a comprehensive approach are required for HD patients. Patients should be encouraged to actively participate in self-management of their disease. Empowerment programs that focus on increasing awareness, knowledge, skills, motivation, self-esteem and the creation of self-efficacy in self-control and preventive behaviors will lead to increases in self-care self-efficacy, QoL, and H&H, a decrease in IDWG, and stabilization of blood pressure. However due to the small sample size of this study our conclusions are not definitive. Further studies with a larger sample size and evaluation of the long term effects of such programs are recommended.

## Abbreviations

(ESRD): End stage renal disease; (CRF): Chronic renal failure; (QoL): Quality of life; (Na^+^): Sodium; (K^+^): Potassium; (Cr): Creatinine; (BUN): Blood urea nitrogen; (P): Phosphorous; (Ca^+^): Calcium; (H&H): Hemoglobin and hematocrit; (SUPPH): Strategies used by people to promote health; (HD): Hemodialysis; (IDWG): Interdialytic weight gain.

Name of the institution where the work was performed: Shiraz University of Medical Sciences (SUMS), Shiraz, Iran

## Competing interests

The authors declare that they have no competing interests.

## Authors’ contributions

MM devised the concept for the study, developed the study design, supervised data collection and analysis, drafted the manuscript, and was involved in study coordination and manuscript revision. ME collected data, ran the study intervention, was involved in the conception of the study, performed the analyses and drafted the manuscript. NS contributed to the study design and intervention. JR contributed to the design and provided feedback. All authors read and approved the final manuscript.

## References

[B1] Wu AW Fink NE Marsh-Manzi JV Meyer KB Finkelstein FO Chapman MM Powe NR Changes in Quality of Life during Hemodialysis and Peritoneal Dialysis Treatment: Generic and Disease Specific Measures J Am Soc Nephrol 2004 15 3 743 753 10.1097/01.ASN.0000113315.81448.CA 14978177

[B2] Xue JL Ma JZ Louis TA Collins AJ Forecast of the number of patients with End-Stage Renal Disease in the United States to the year 2010 J Am Soc Nephrol 2001 12 12 2753 2758 11729245 10.1681/ASN.V12122753

[B3] Rambod M Rafei F Hossini F Quality of life in patients with chronic renal failure Journal of Nursing and Midwifery Faculty of Tehran University of Medical Sciences (Hayat) 2008 14 2 51 61 Persian

[B4] Zamanzadeh V Heidar Zadeh M Ashvandi K lak dizaji S The relationship between quality of life and social support in hemodialysis patients Medical Journal of Tabriz University of Medical Sciences 2007 29 1 49 54 Persian

[B5] Curtin RB Sitter DC Schatell D Chewning BA Self-management, knowledge, and functioning and well-being of patients on hemodialysis Nephrol Nurs J 2004 31 4 378 386 15453230

[B6] Sajjadi M Kushyar H Vaghei S Ismeili HA The effect of education on depression in patients treated with hemodialysis Journal of Birjand University of Medical Sciences 2008 15 1 34 40 Persian

[B7] Tsay SL Healstead M Self-care self-efficacy, depression, and quality of life among patients receiving hemodialysis in Taiwan. Int J Nurs Stud 2002 39 3 245 251 10.1016/S0020-7489(01)00030-X 11864647

[B8] Curtin RB Walters BA Schatell D Pennell P Wise M Klicko K Self-Efficacy and self-management behaviors in patients with chronic kidney disease Adv Chronic Kidney Dis 2008 15 2 191 205 10.1053/j.ackd.2008.01.006 18334246

[B9] Heidari M Alhani F Kazemnejad A Moezzi F The effect of empowerment model on quality of life of Diabetic adolescents Iran J Pediatr 2007 17 1 87 94 Persian

[B10] Funnell MM Anderson RM Empowerment and self-management of diabetes Clinical Diabetes 2004 22 3 123 127 10.2337/diaclin.22.3.123

[B11] Thomas-Hawkins C Zazworsky D Self-management of chronic kidney disease AJN 2005 105 10 40 48 10.1097/00000446-200510000-00030 16205407

[B12] Moattari M Ghobadi A Beigi P Pishdad G Impact of self management on metabolic control indicators of diabetes patients Journal of Diabetes & Metabolic Disorders 2012 11 6 10.1186/2251-6581-11-6 PMC3581103 23497728

[B13] Funnell MM Anderson RM Robert M From DSME to DSMS: Developing Empowerment-Based Diabetes Self-Management Support. Diabetes Spectrum 2007 20 4 221 226

[B14] Naccashian Z The impact of diabetes self-management education on glucose management in ethnic Armenians with type 2 diabetes September 8, 2011 [Paperback] ProQuest, UMI Dissertation Publishing 10.1177/0145721714535993 24872385

[B15] Moattari M Hashemi M Dabbaghmanesh MH The impact of electronic education on metabolic control indicators in patients with diabetes who need insulin: a randomised clinical control trial J Clin Nurs 2012 Epub ahead of print PMID:22905971 10.1111/j.1365-2702.2012.04200.x 22905971

[B16] Hughes J Wood E Smith G Exploring kidney patients’ experiences of receiving individual peer support Health Expect 2009 12 4 396 406 10.1111/j.1369-7625.2009.00568.x 19691464 PMC5060506

[B17] Rantanen M Kallio T Johansson K Salanterä S Virtanen H Leino-Kilpi H Knowledge expectations of patients on dialysis treatment Nephrol Nurs J 2008 35 3 249 255 18649585

[B18] Birmelé B Le Gall A Sautenet B Aguerre C Camus V Clinical, sociodemographic, and psychological correlates of health-related quality of life in chronic hemodialysis patients Psychosomatics 2012 53 1 30 37 10.1016/j.psym.2011.07.002 22221719

[B19] Mason J Khunti K Stone M Farooqi A Carr S Educational interventions in kidney disease care: a systematic review of randomized trials Am J Kidney Dis 2008 51 6 933 951 10.1053/j.ajkd.2008.01.024 18440681

[B20] Tasy Sh L Hung LO Empowerment of patients with end-stage renal disease–A randomized controlled trial Int J Nurs Stud 2004 41 59 65 10.1016/S0020-7489(03)00095-6 14670395

[B21] Chen Y-C Pai J-S Li I-C Hemodialysis: the effects of using the empowerment concept during the development of a mutual-support group in Taiwan Journal compilation 2008 17 5A 133 142 10.1111/j.1365-2702.2007.02186.x 18298764

[B22] Zimmerman MA Psychological empowerment: issues and illustrations Am J Community Psychol 1995 23 5 581 599 10.1007/BF02506983 8851341

[B23] Bennett PN Satellite dialysis nursing: technology, caring and power J Adv Nurs 2011 67 1 149 157 10.1111/j.1365-2648.2010.05474.x 20955185

[B24] Lev Elise L Self-efficacy in patients with prostate cancer: Confirmatory factor analysis of Strategies Used by Patients to Promote Health. The 18th Annual Scientific Sessions of the Eastern Nursing Research Society, New Momentum for Nursing Research: Multidisciplinary Alliances, April 21, 2006 2006 http://hdl.handle.net/10755/163209

[B25] Lev EL Owen SV Confirmatory factor analysis for SUPPH.A presentation in a symposium,“ Definitions,measurement,theoretical basis and relationships associated with self care self-efficacy”13 Annual scientific sessions of the eastern nursing research association 2001 Atlantic City,NJ http://hdl.handle.net/10755/163780

[B26] http://www.uic.edu/orgs/qli/questionaires/pdf/genericversionIII/generic.pdf

[B27] Ferrans CE Powers MJ Quality of life index: development and psychometric properties ANS Adv Nurs Sci 1985 8 1 15 24 3933411 10.1097/00012272-198510000-00005

[B28] Ferrans CE Powers MJ Psychometric assessment of the Quality of Life Index Res Nurs Health 1992 15 1 29 38 10.1002/nur.4770150106 1579648

[B29] Agarwal R Management of hypertension in hemodialysis patients Hemodial Int 2006 10 241 248 10.1111/j.1542-4758.2006.00102.x 16805884

[B30] Norman GR Sloan JA Wyrwich KW Interpretation of Changes in Health-Related Quality of Life Medical Care 2003 41 5) 582 592 12719681 10.1097/01.MLR.0000062554.74615.4C

[B31] Goldsmith CH Boers M Bombardier C Tugwell P Criteria for clinically important changes in outcomes: development, scoring and evaluation of rheumatoid arthritis patient and trial profiles OMERACT Committee J Rheumatol 1993 20 3 561 565 8478874

[B32] Esmaeili M Alikhani M Khollam Araghei M Hossini F Quality of life and relationship with self-efficacy in patients under hemodialysis Journal of Nursing 2006 18 41,42 78 84 Persian

[B33] DeOreo PB Hemodialysis patient-assessed functional health status predicts continued survival, hospitalization, and dialysis-attendance compliance Am J Kidney Dis 1997 30 2 204 212 10.1016/S0272-6386(97)90053-6 9261030

[B34] Merkus MP Jager KJ Dekker FW De haan RJ Boeschoten EW Krediet RT Physical symptoms and quality of life in patients on chronic dialysis: results of The Netherlands Cooperative Study on Adequacy of Dialysis (NECOSAD) Nephrol Dial Transplant 1999 14 1163 1170 10.1093/ndt/14.5.1163 10344356

[B35] Diaz-Buxo JA Lowrie EG Lew NL Zhang H Michael Lazarus J Quality-of-life evaluation using Short Form 36: comparison in hemodialysis and peritoneal dialysis patients Am J Kidney Dis 2000 35 2 293 300 10.1016/S0272-6386(00)70339-8 10676729

[B36] Walters BA Hays RD Spritzer KT Fridman M Carter WB Health-related quality of life, depressive symptoms, anemia and malnutrition at hemodialysis initiation Am J Kidney Dis 2002 40 6 1185 1194 10.1053/ajkd.2002.36879 12460037

[B37] Huang IC Liu JH Wu AW Wu MY Leite W Hwang CC Evaluating the reliability, validity and minimally important difference of the Taiwanese version of the diabetes quality of life (DQOL) measurement Health Qual Life Outcomes 2008 28 6 87 10.1186/1477-7525-6-87 PMC2603003 18957127

[B38] Rodwell CM An analysis of the concept of empowerment J Adv Nurs 1996 23 2 305 313 10.1111/j.1365-2648.1996.tb02672.x 8708244

[B39] Mitcheson J Cowley S Empowerment or control? An analysis of the extent to which client participation is enabled during health visitor/client interactions using a structured health needs assessment tool Int J Nurs Stud 2003 40 4 413 426 10.1016/S0020-7489(02)00107-4 12667518

[B40] Lii Y-C Tsay S-L Wang T-S Group intervention to improve quality of life in hemodialysis patients Journal of nursing and healthcare of chronic illness in association with Journal of Clinical Nursing 2007 16 11 268 275 10.1111/j.1365-2702.2007.01963.x 17931320

[B41] Thomas D Joseph J Francis B Mohanta GP Effect of patient counseling on quality of life of hemodialysis patients in India Pharmacy Practice (Internet) 2009 7 3 181 184 10.4321/s1886-36552009000300009 PMC4139051 25143797

[B42] Baraz S Mohammadi E Bromand B Effect of diet education on the laboratory indicators and interdialytic weight gains in patients treated with maintenance hemodialysis Shahrecord School of Medical Sciences journal 2006 8 1 20 27 Persian

[B43] Saint-Remy A Krzesinski JM Optimal Blood Pressure Level and Best Measurement Procedure in Hemodialysis Patients Vasc Health Risk Manag 2005 1 3 235 244 17319109 PMC1993946

[B44] Aujoulat I d’Hoore W Deccache A Patient empowerment in theory and practice: Polysemy or cacophony? Patient Educ Couns 2007 66 1 13 20 10.1016/j.pec.2006.09.008 17084059

[B45] Agarwal R Peixoto AJ Santos SF Zoccali C Pre and post dialysis blood pressures are imprecise estimates of interdialytic ambulatory blood pressure Clin J Am Soc Nephrol 2006 1 3 389 398 10.2215/CJN.01891105 17699236

[B46] Sinha Arjun D Agarwal R Peridialytic, Intradialytic, and Interdialytic Blood Pressure Measurement in Hemodialysis Patients America Journal of Kidney Diseases 2009 54 5 788 79 10.1053/j.ajkd.2009.07.004 PMC2784813 19853196

[B47] Inrig JK Patel UD Toto RD Szczech LA Association of Blood Pressure Increases During Hemodialysis With 2-Year Mortality in Incident Hemodialysis Patients: A Secondary Analysis of the Dialysis Morbidity and Mortality Wave 2 Study Am J Kidney Dis 2009 54 5 881 890 10.1053/j.ajkd.2009.05.012 19643520 PMC2767411

[B48] Kugler C Maeding I Russell CL Non-adherence in patients on chronic hemodialysis: an international comparison study J NepAhrol 2011 24 3 366 375 10.5301/JN.2010.5823 20954134

[B49] Holmberg B Stegmayr BG Cardiovascular conditions in hemodialysis patients may be worsened by extensive interdialytic weight gain Hemodial Int 2009 13 1 27 31 10.1111/j.1542-4758.2009.00335.x 19210274

[B50] Davenport A Cox C Thuraisingham R PanThames Renal Audit Group The importance of dialysate sodium concentration in determining interdialytic weight gains in chronic hemodialysis patients: The PanThames Renal Audit Int J Artif Organs 2008 31 5 411 417 18609514 10.1177/039139880803100506

[B51] Russell CL Cronk NJ Herron M Knowles N Matteson ML Peace L Ponferrada L Motivational Interviewing in Dialysis Adherence Study (MIDAS) Nephrol Nurs J 2011 38 3 229 236 21877456

[B52] Singh AK The FDA Black Box for EPO: what should nephrologists do? Nephrol News Issues 2007 21 6 55 56 58-59 17518125

[B53] Singh AK Milford E Fishbane S Keithi-Reddy SR Managing anemia in dialysis patients: hemoglobin cycling and overshoot Kidney Int 2008 74 5 679 683 10.1038/ki.2008.59 18337717

[B54] Moreno F Sanz-Guajardo D López-Gómez JM Jofre R Valderrábano F Increasing the hematocrit has a beneficial effect on quality of life and is safe in selected hemodialysis patients. Spanish CooperativAe Renal Patients Quality of Life Study Group of the Spanish Society of Nephrology J Am Soc Nephrol 2000 11 2 335 342 10665941 10.1681/ASN.V112335

